# Taggart Suit Decided

**Published:** 1913-03-15

**Authors:** 


### TAGGART SUIT DECIDED.

EDITOR DENTAL REGISTER.—DEAR DOCTOR: The National Dental Protective Association sends you greet ingand announces that it has won the final decree in the Court of Appeals against Taggart in the Taggart-Boynton suit. Enclosed is copy of the *Decision*, also our By-Laws. Will you help to get a thousand new members in an Association which can protect its members as demonstrated by this decision. The fund so raised is needed to meet the expenses incurred. Our attorneys have not been paid, as they took the case on a contingent fee and are now entitled to their pay.

M. F. FINLEY, 1928 Eye St. N. W., *Secy.-Treas.*

E. P. DAMERON, St. Louis. *President.*

WASHINGTON, D. C., *March 1, 1913.*

